# DSM-5 Criteria and Depression Severity: Implications for Clinical Practice

**DOI:** 10.3389/fpsyt.2018.00450

**Published:** 2018-10-02

**Authors:** Julio C. Tolentino, Sergio L. Schmidt

**Affiliations:** Department of Neurology, Federal University of the State of Rio de Janeiro, Rio de Janeiro, Brazil

**Keywords:** depression, cardiovascular system, autonomic nervous system, suicide, sudden death, cardiac arrhythmias

## Abstract

**Background:** Depression diagnosis requires five or more symptoms (Diagnostic and Statistical Manual of Mental Disorders-DSM-5). One of them must be either Depressed mood or Anhedonia, named main criteria. Although the secondary symptoms can be divided into somatic and non-somatic clusters, the DSM-5 identify depression in all or none fashion. In contrast, depression severity is a continuous variable. Therefore, it is commonly assessed with scales such as the Hamilton Depression Rating Scale (HAMD). Previously, we reported that patients with moderate depression (MD) exhibit greater impairments in cardiac-autonomic modulation than severely depressed (SD) patients. However, clinicians usually do not use scales.

**Objective:** To verify whether the DSM-5 symptoms would be able to discriminate SD from MD and MD from non-depressed (ND) subjects.

**Material and Methods:** Depression was diagnosed based on the Structured Clinical Interview for DSM-5® Disorders. The HAMD evaluated depression severity. In depressed subjects, MD and SD were defined considering the HAMD scores. ND was defined considering both the absence of DSM-5 criteria for depression and the HAMD score. Among 782 outpatients, 46 SD were found. MD and ND subjects were randomly sampled to match the demographic variables of the SD group.

**Results:** Discriminant analysis showed that Depressed Mood was the most reliable symptom to discriminate ND from MD. Anhedonia discriminated SD from MD. Among the secondary DSM-5 criteria, the somatic cluster discriminated ND from MD and the non-somatic cluster SD from MD patients.

**Discussion:** The presence of the somatic cluster in MD may indicate decreased vagal tone and/or increased sympathetic tone, leading to higher cardiovascular risk. As SD is associated with the non-somatic cluster, these patients are at risk of committing suicide. The DSM-5 symptoms exhibited by the patient may help the choice of adequate pharmacological treatment. This would avoid the use of antidepressants that unnecessarily increase cardiac risk in MD. When the symptom cluster suggests SD, the treatment must focus on the prevention of suicide.

**Conclusions:** Depression severity may be inferred based on the DSM-5 criteria. The presence of the Anhedonia main criterium accompanied by non-somatic criteria indicate SD. The Depressive Mood criterium followed by somatic criteria suggest MD.

## Introduction

Depression is a common psychiatric disorder, with an estimated lifetime prevalence of 10% in the general population (1, 2). In clinical settings, its prevalence may reach as high as 20% (1, 3). According to the Diagnostic and Statistical Manual of Mental Disorders, Fifth Edition (DSM-5), the diagnosis of a Major Depression Episode (MDE) requires five or more symptoms to be present within a 2-week period (4). One of the symptoms should, at least, be either a depressed mood (DM) or anhedonia (loss of interest or pleasure- LI). The secondary symptoms of MDE are appetite or weight changes (AW), sleep difficulties (insomnia or hypersomnia), psychomotor agitation or retardation (PAR), fatigue or loss of energy (FE), diminished ability to think or concentrate (C), feelings of worthlessness or excessive guilt (FW), and suicidality (SU). These symptoms are rated in an all or none (0 or 1) fashion.

According to the DSM-5 criteria, the symptoms are summed to determine the presence or the absence of a major depression episode (4). Consequently, the DSM assumes that the depression construct may be considered unidimensional. However, several studies have described different subtypes of depression (1, 5, 6). Furthermore, the unidimensional model of depression has been challenged by studies on the factor structure of the DSM symptom criteria (7–9). Elhai et al. (10) have reported that a two-factor model fits better than the one-factor unidimensional mode. They found that major depression symptoms are best represented by somatic and non-somatic factors. The somatic items included sleep difficulties (SD), appetite or weight changes, poor concentration, fatigue, and psychomotor agitation/retardation. The non-somatic factor consisted of affective items such as depressed mood, anhedonia, feelings of worthless, and thoughts of death.

Previous investigations have reported that cognitive dysfunction, age, psychosis, unemployment, suicide ideation are associated with depression severity (11–13). However, to our knowledge, there is a lack of a systematic study on the relationship between DMS-5 symptoms and depression severity. There is no consensus if the number of symptoms is indicative of depression severity or even if the degree of each symptom can be used as an index to classify depression as mild, moderate, or severe. Consequently, the severity of depression is commonly assessed with the aid of rating depression scales, such as the Hamilton Depression Rating Scale (HAMD) (14). HAMD has been the most frequently used rating scale for depression (14–17).

Almas et al. (18) reported that moderately depressed persons showed a higher risk for cardiovascular disease (CVD) compared to severely depressed patients. Recently, Tolentino and Schmidt (19) have shown that cardiac autonomic regulation is associated with depression severity, as measured by the HAMD, in an intriguing way: moderately depressed patients showed greater impairment in autonomic modulation as compared to either severely depressed patients or non-depressed subjects. Then, subjects with moderate depression who do not seek treatment are at higher risk of CVD. However, some antidepressants such as the tricyclics increase the risk of cardiac arrhythmia and sudden cardiac death (20–23). Therefore, structured and unstructured clinical interviews need to be supplemented by ratings based on appropriated scales (24). As sometimes the use of extensive scales is not possible, there is a practical interest to verify whether depression severity could be assessed using the DSM5 symptoms.

The present study aimed to verify whether the DSM-5 criteria for depression would be able to discriminate moderate from severe depression as assessed with the aid of the HAMD. A clinical sample was selected because of the highest prevalence of depression in this population (1, 2). Based on the effects of moderate depression on cardiac autonomic modulation (16), we hypothesized that specific somatic DSM-5 symptoms might be closely related to moderate depression. Symptom expressions such as sleep difficulties, appetite or weight changes, and psychomotor agitation/retardation would be maximized in moderately depressed patients as compared to non-depressive subjects. In addition, patients with severe depression would be characterized by thoughts of death and feelings of worthlessness.

## Materials and methods

### Study sample

**Figure 1:**
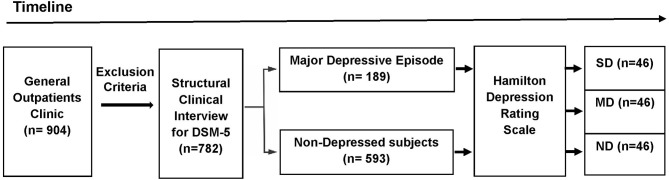
Design of the study (timeline). The initial sample consisted of 904 outpatients. After applying the exclusion criteria, 782 eligible patients were recruited to participate in first part of this study. Based on the DSM-5 criteria, 189 patients exhibited a major depressive episode at the time of the Structured Clinical Interview for DSM-5® Disorders-Clinician Version. The Hamilton Depression Rating Scale (HAMD) were administered in the depressed outpatients and the non-depressed group (*n* = 593). Based on the HAMD cut-off scores, three groups were selected: Non-depressed (ND), Moderately Depressed (MD), and Severely Depressed (SD). After the analysis of the HAMD scores (*n* = 782), 46 severely depressed patients were found. Age, percentage of females, years of education, and mini-mental state exam scores were calculated for the SD group. These demographic values were used to sample the other two groups (ND and MD). Then, from the total number of subjects in the other two groups (ND and MD), 92 subjects (46 MD and 46 ND) were randomly selected to be included in the analyses.

The participants were selected from a primary care practice located at Rio de Janeiro, Brazil (Gaffrée and Guinle University Hospital). The research protocol was administered by well-trained raters. The initial sample consisted of 904 outpatients. The exclusion criteria were: age below 18 years; patients with neurological diseases; alcohol and substance use-related disorders; dementia and other cognitive disorders (Mini-Mental State Examination score below 24 points); presence of psychotic symptoms; uncontrolled clinical diseases, such as hypothyroidism, diabetes, or hypertension; and who were taking antidepressants within the last 3 months. After applying the exclusion criteria, 782 eligible patients were recruited to participate in first part of this study. Based on the Structured Clinical Interview for DSM-5® Disorders-Clinician Version (SCID-5-CV) (25), 189 patients were found to exhibit MDE at the time of the interview. After the analysis of the HAMD scores (*n* = 782), 46 severely depressed (SD) patients were found. The participants in the Non-depressed and Moderately depressed groups were selected based on matching demographic characteristics with the Severely depressed group. Then, 46 controls and 46 moderately depressed patients were randomly selected to be analyzed (Figure 1). The non-depressed group was composed of patients without criteria for depression through SCID-5-CV and HAMD.

### Procedures

The Mini-Mental State Examination (26) was used to assess cognitive function in each general outpatients clinic (*n* = 904). Psychiatric assessments were done in 782 subjects included in the study, through the application of SCID-5-CV to MDE diagnosis and the 17-items of the Hamilton Depression Rating Scale (17-item HAMD) to measure depression severity (14, 17, 27, 28). The SCID-5-CV that was applied for the MDE diagnosis is a semi-structured interview for making DSM-5 diagnoses (25). It was administered by trained physicians that are familiar with the DSM-5 diagnostic criteria.

The 17-item, clinician-administered Hamilton Rating Scale for Depression was administered in the depressive outpatients (*n* = 189) and the non-depressed group (*n* = 583). Two trained raters independently scored each patient at the same interview. Only patients who received equal scores from the two raters were included in the sample. It should be mentioned that high interrater reliability has been described previously (16, 28, 29). The total score on the 17-item HAMD ranges from 0 to 52, with higher scores representing greater severity of depression. In this study, the 17-item HAMD cut-off points were defined as follows: >24 = severe; 17–23 = moderate; 8–16 = mild; and none (non-depressed) = 0–7 (17). Based on these HAMD cut-off scores, three groups were selected: Non-depressed (ND), Moderately Depressed (MD), and Severely Depressed (SD).

### Ethical approval

The study is consistent with the declaration of Helsinki, and it was approved by Gaffrée and Guinle University Hospital Ethics committee. All subjects gave written informed consent in accordance with the Declaration of Helsinki.

### Statistical analysis

Quantitative variables are reported as absolute and relative frequencies, means (*M*), and standard deviations. Across the demographic variables, group differences were tested using *T-*tests for the continuous variables and *chi-square* tests (χ^2^) for the categorical variables (Table 1).

**Table 1:** Comparison of matched groups variables according to the Hamilton Depression Rating Scale.

	**Non-depression**	**Moderate depression**	**Severe depression**	**ND vs. MD**	**MD vs. SD**
	**(*n* = 46)**	**(*n* = 46)**	**(*n* = 46)**	***p***	***p***
Age (years), mean (standard deviation)	46.5 (14.5)	46.2 (14.4)	45.8 (13.6)	0.91	0.87
					
Female sex, number (%)	33 (71.7%)	33 (71.7%)	33 (71.7%)	1.0	1.0
Education (years), mean (standard deviation)	9.7 (4.8)	9.1 (3.4)	8.8 (4.3)	0.37	0.32
Mini-mental status examination (score), mean (standard deviation)	28.1 (2.1)	27.9 (1.7)	27.4 (2.5)	0.47	0.21
Hamilton depression rating scale (score), mean (standard deviation)	2.5 (1.4)	19.7 (2.1)	28.9 (3.7)	<0.001	<0.001

*ND, Non-depressed group; MD, Moderately depressed group; SD, Severely depressed group; p, proof value*.

Discriminant analysis was performed to examine if the DSM-5 criteria accurately distinguished between Non-depressed, Moderately Depressed and Severely Depressed groups as defined by the 17-item HAMD scores. Results will be presented for Non-depressed vs. Moderately Depressed, and Severely Depressed vs. Moderately Depressed.

Initially, the equality of the group means was tested using Wilk's λ. It should be mentioned that the smaller the λ, the more important is the independent variable to the discriminant function. Then, the assumptions of the discriminant analyses were tested (linearity, normality, multilinearity, equal variances, and multivariate normal distribution of the predictors). Box's *M*-tests were performed to test the assumption of the homogeneity of covariance matrices. It should be mentioned that discriminant analysis is robust when the homogeneity of variances assumption is not met, provided the data do not contain important outliers. For our data, the Box's *M*-test was interpreted in conjunction with the inspection of the log determinants. Considering our sample size and the absence of outliers, we concluded that the small deviations from homogeneity groups did not violate the assumptions of the discriminant analysis.

For each case (Non-depressed vs. Moderately Depressed and Severely Depressed vs. Moderately Depressed), the discriminant was created as a linear combination of the nine independent variables. The standardized canonical coefficients of the discriminant function analysis were used to identify the most reliable variable for discriminating between Severely Depressed and Moderately Depressed groups as well as between Non-depressed and Moderately Depressed groups. The Pearson correlations between predictors and standardized canonical discriminant functions were calculated and loadings <0.30 were removed from the model. Then, the canonical discriminant function coefficients were calculated to obtain the Discriminant Function (DF). Canonical correlations (λs) were calculated to measure how well each DF separate cases into the two groups (Moderately Depressed vs. Non-depressed, and Moderately Depressed vs. Severely Depressed). For each case, the correspondent chi-squared was calculated to verify if the DF did better than the chance level of separating the two groups. With the aid of the DF, the accuracy of the classification was measured for each case.

SPSS Statistics for Windows, version 22.0 (SPSS Inc., Chicago, IL) was used for analysis, and the significance level was set at *p* < 0.0.5.

## Results

The age ranged from 21 to 82 years (*M* = 45.9; standard deviation = 14.6) in the sample selected to participate in the first part of the study (eligible outpatients; *n* = 782). Most participants were female sex (65.3%). The mean years of schooling and the Mini-Mental State Examination (MMSE) scores were 9.1 (standard deviation = 4.1) and 28.1 (standard deviation = 2.6), respectively. The prevalence of MDE in this clinical sample was 24.2% (*n* = 189), and the mean score on the HAMD was 9.7 (standard deviation = 2.8).

In the whole sample, 46 depressive patients were found to be severely depressed. Severe depression was found to be more frequent in women (71.7%). Logistic regression showed that female sex was associated with severe depression (odds ratio = 3.74; 95% confidence interval = 1.66–8.42). In contrast, there was not an association between sex and moderate depression.

The Non-depressed and the Moderately Depressed groups were matched considering the demographic variables of the Severely Depressed group (Table 1). There was no statistically significant differences according to race (Caucasians and non-Caucasians) among the three groups. The human development index in our sample ranged from 0.782 to 0.842 and no differences were found among the three groups. The relative frequency distributions of the nine DSM-5 criteria for each group indicated that patients with severe depression exhibited more non-somatic DSM-5 symptoms than moderately depressed patients, especially for anhedonia, feelings of worthlessness/excessive guilt, and suicidality (Table 2). As expected, all the DSM-5 symptoms were found to be more frequent in Severely Depressed and Moderately Depressed groups as compared to the control (Non-depressed) group.

**Table 2:** The relative frequencies of the DSM-5 criteria for major depressive episode among the groups.

	**DM**	**LI**	**AW**	**SD**	**PAR**	**FE**	**FW**	**C**	**SU**
Severely depressed, number (%)	46 (100%)	41 **(89.1%)**	36 (78.3%)	45 (97.8%)	34 (73.9%)	43 (93.5%)	35 **(76.1%)**	39 (76.1%)	29 **(63%)**
Moderately depressed, number (%)	42 (91.5%)	30 **(65.2%)**	30 (65.2%)	43 (93.5%)	32 (69.6%)	43 (93.5%)	24 **(52.2%)**	35 (76.1%)	13 **(28.3%)**
Non-depressed, number (%)	5 (10.9%)	3 **(6.5%)**	4 (8.7%)	3 (6.5%)	4 (8.7%)	11 (23.9%)	2 **(4.3%)**	4 (8.7%)	2 **(4.3%)**

*DM, depressed mood; LI, loss of interest or pleasure; AW, appetite or weight disturbance; SD, sleep difficulties (insomnia or hypersomnia); PAR, psychomotor agitation or retardation; FE, fatigue or loss of energy; FW, feelings of worthlessness or excessive guilt; C, diminished ability to think or concentrate; SU, suicidality. Note the markedly differences among Severely Depressed group and the other two groups for LI, FW, and SU (bold)*.

### Non-depressed group vs. moderately depressed group

Group means were found to be significantly different for all DSM-5 criteria. The smallest Wilk's λ was found for depressed mood followed by sleep difficulties (insomnia or hypersomnia). Depressed mood was the most reliable variable for discriminating between groups, followed by sleep difficulties and poor concentration. The smallest discriminant ability was found for suicidality. The pooled within-groups correlations (Table 3) identified the large correlations with the full discriminant model: Depressed mood, sleep difficulties, poor concentration, and fatigue. The lowest was suicidality followed by feelings of worthlessness/excessive guilt. After excluding loadings <0.30, the following discriminant function (DF) was deduced from the analysis: DF = −3.290 + (2.120^*^DM) + (2.40^*^SD) + (0.764^*^FE) + (1.327^*^C).The canonical discriminate function reached an eigenvalue of 6.931 (χ^2^ = 182.23, *d.f* = 4, *p* < 0.001). Therefore, the DF significantly separated the two groups. Based on the DF formula, subjects with DF > 0 were classified as moderately depressed, and subjects with DF < 0 were classified as controls with 98% accuracy.

**Table 3:** Pooled within-groups correlations between discriminating variables and standardized canonical discriminant function: Non-depression vs. moderate depression, and Moderate depression vs. Severe depression.

**Non-depression vs. moderate depresssion-variables**	**Loadings**
Depressed mood	0.48
Insomnia or hypersomnia	0.48
Poor concentration	0.34
Fatigue or loss of energy	0.31
Loss of interest or pleasure	0.27
Appetite or weight disturbance	0.26
Psychomotor agitation or retardation	0.26
Feelings of worthlessness or excessive guilt	0.21
Suicidality	0.12
**Moderate depression vs. severe depression-variables**	**Loadings**
Suicidality	0.64
Loss of interest or pleasure	0.51
Feelings of worthlessness or excessive guilt	0.44
Depressed mood	0.37
Appetite or weight disturbance	0.25
Poor concentration	0.19
Insomnia or hypersomnia	0.18
Psychomotor agitation or retardation	0.08
Fatigue or loss of energy	0.00

*Correlations < 0.30 were excluded from the discriminant equation*.

### Moderately depressed group vs. severely depressed group

Group means were found to be significantly different for depressed mood, loss of interest or pleasure (anhedonia), feelings of worthlessness/excessive guilt, and suicidality. The smallest Wilk's λ was found for suicidality followed by anhedonia. The highest value was fatigue. The analysis of the standardized canonical coefficients indicated that suicidality was the most reliable variable for discriminating between the groups, followed by anhedonia. The smallest discriminant ability was found for fatigue. The pooled within-groups correlations (Table 3) identified the large correlations with the DF (suicidality, loss of interest or pleasure, feelings of worthlessness/excessive guilt, and depressed mood). The lowest was fatigue.

The following discriminant function (DF) was deduced from the analysis: DF = −3.078 + (1.147^*^LI) + (0.596^*^FW) + (1.404^*^SU) + (1.222^*^DM).

The canonical discriminate function reached an eigenvalue of 0.261(χ^2^ = 20.44, *d.f* = 4, *p* < 0.001). Therefore, the DF significantly separated the two groups. Based on the DF formula, subjects with DF > 0 were classified as Severely Depressed and subjects with DF < 0 were classified as Moderately Depressed with 72.7% accuracy.

**Figure 2:**
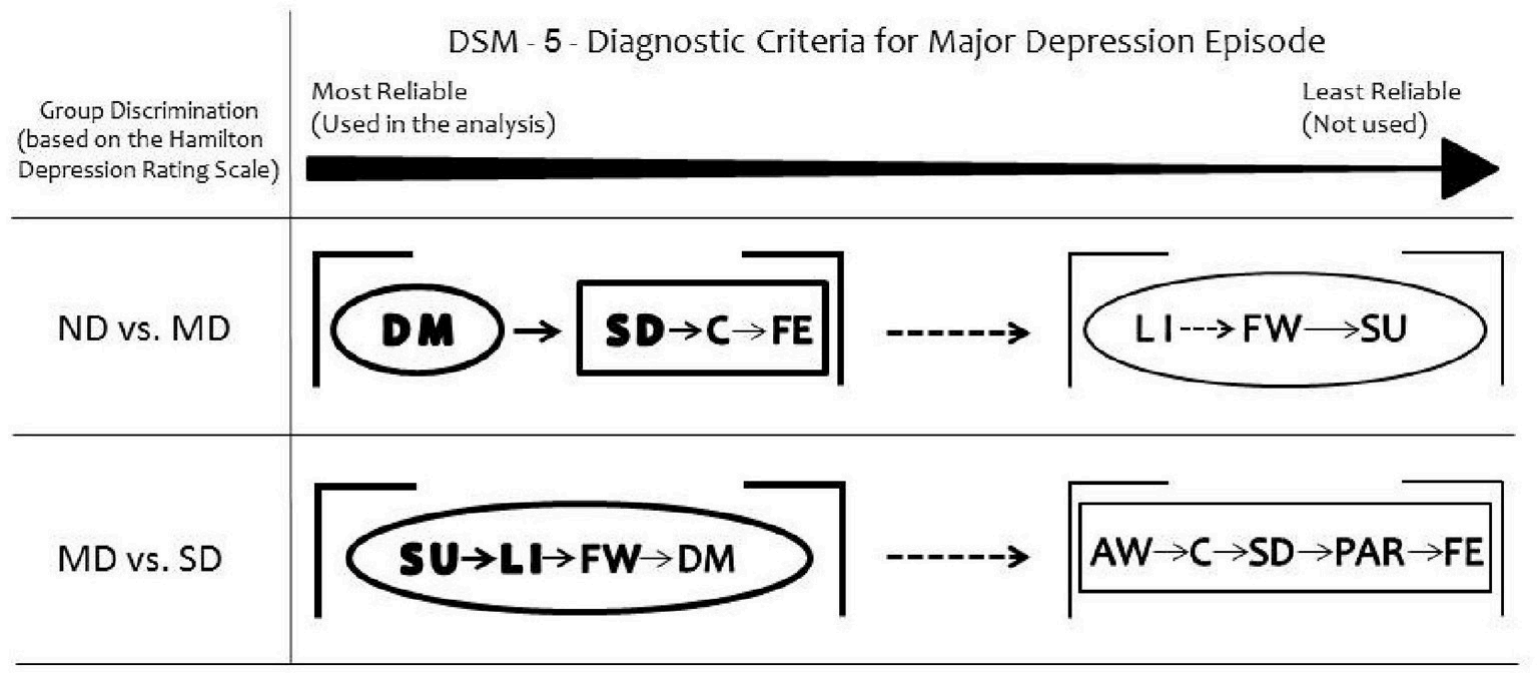
Summary of the results. Depressed mood is the most reliable DSM-5 symptom to discriminate moderately depressed (MD) group from non-depressed (ND) group. Loss of interest or pleasure discriminates severely depressed (SD) group from MD. Considering the secondary DSM-5 criteria, the somatic items discriminate MD from ND groups. All the non-somatic DSM-5 criteria separate MD from SD groups. The ellipses represent the non-somatic DSM-5 items and the rectangles the somatic DSM-5 items, according to the factor structure described by Elhai et al. (10). DM, depressed mood; LI, loss of interest or pleasure; SD, sleep difficulties (insomnia or hypersomnia); C, diminished ability to think or concentrate; FE, fatigue or loss of energy; FW, feelings of worthlessness or excessive guilt; SU, suicidality; AW, appetite or weight disturbance; PAR, psychomotor agitation or retardation.

Taken together, the somatic DSM-5 items discriminated Moderately Depressed from Non-depressed and all the affective items discriminated Moderately Depressed from Severely Depressed groups (Figure 2).

## Discussion

The two main diagnostic criteria for depression (depressed mood and loss of interest or pleasure) differ regarding their discrimination ability when the level of depression is considered: depressed mood is the most reliable DSM-5 symptom to discriminate moderate depression from non-depression whereas anhedonia emerges as an important criterion when depression becomes more severe. Among the secondary DSM criteria, the somatic cluster shows high discriminant ability to separate non-depression from moderate depression. For the discrimination of severe from moderate depression, the most reliable DSM-5 symptom is suicidality, followed by anhedonia, feelings of worthlessness and depressed mood. In summary, the non-somatic DSM-5 criteria are found to distinguish moderate from severe depression reliably, while the somatic factors are useful for the discrimination between moderate and non-depression groups.

The present data support a two-factor model of depression proposed by Elhai et al. (10). Among the secondary DSM-5 symptoms, the somatic factors are related to moderate depression, whereas the non-somatic or cognitive-affective factors are related to severe depression. The finding that the two main criteria for depression (depressed mood and anhedonia) exhibit distinct discrimination ability may reflect the possible differences between these two symptoms. Depressive mood is often associated with the presence of stressors (30), often loss situations (death, economic reversal, separation, illness, etc.). Thus, it is possible to speculate that depressed mood may be considered a compound factor indicating either a response to stressful situations (somatic factor) or a sadness feeling (affective factor). Anhedonia may indicate either loss of interest (motivational anhedonia or absence of an anticipatory pleasure from future activities) or loss of pleasure in response to stimuli that were previously perceived as rewarding (consummatory anhedonia) (31). Therefore, anhedonia is fully related to the affective factor while depressed mood might be related to both affective and somatic factors (stressful situations). This might explain why depressed mood is a good discriminator of moderate from non-depressed groups. However, another interpretation is that HAMD simply gives more importance to depressed mood items than to anhedonia (14, 16, 17).

Depression has been closely associated with autonomic nervous system dysfunction, with reduced parasympathetic and/or increased sympathetic activity leading to increased cardiovascular risk in depressed patients (32–34). In line with these findings, increased inter-lead QT interval differences on 12-lead electrocardiography (QT dispersion) or reduced heart rate variability (HRV) has been reported in depressed patients (19, 35–41). Either reduced HRV or augmented QT dispersion reflects excessive sympathetic modulation and/or inadequate cardiac vagal control (19, 35–44). Thus, both conditions may predispose individuals with depression to ventricular tachycardia, ventricular fibrillation, myocardial ischemia, and sudden cardiac death (44–46). However, research on depression and HRV has been typically conducted in patients with cardiovascular disease (46–50). Our findings are in agreement with the recent report by Benvenuti et al. (51) who showed that somatic depressive symptoms are related to reduced HRV in medically healthy individuals with dysphoria. Here we demonstrated that moderately and severely depressed patients may express symptoms of low mood or distress through two distinct clusters of DSM-5 criteria. The present study report that moderate depression is associated with the somatic cluster is in agreement with the previous finding of highest autonomic dysfunction in moderate depression as compared to all other groups, including control and severely depressed patients (19).

The somatic symptoms may be related to autonomic disturbances in depressed patients without known cardiovascular disease (52, 53). Likewise, the Mental Stress-Induced Myocardial Ischemia has already been described in a patient with normal coronary arteries and generalized anxiety disorder (51). In this case, anxiety might be considered a somatic component of depression (54–57). Decreasing serotonin may cause a decrease in parasympathetic activity (58), and emotional response capabilities are marked peripherally by vagal efference to the heart (40, 59). Specifically, high parasympathetic tone helps to maintain heart stability and protect against possible adverse cardiac events (32, 34). Conversely, increased sympathetic tone increases the risk of malignant arrhythmias and sudden cardiac death (60). Thus, a high degree of HRV provides cardioprotective effect whereas the reduction in HRV is associated with higher cardiovascular risk in depressed patients (43, 51, 52).

Although medication-free depressed patients already exhibit reductions in HRV (52), the use of specific antidepressants (e.g., tricyclics) further decreases HRV (50, 61–65). This poses an additional risk for the depressed patients. The finding that the somatic cluster is related to moderate depression indicates a decrease in parasympathetic activity leading to higher cardiovascular risk. Therefore, the present data suggest that the cluster of DSM-5 symptoms exhibited by the patient may guide the choice of the adequate antidepressant drug treatment.

In addition, severe depression was found to be linked to increased suicidality, highlighting the importance of needing clear markers of severe depression for clinicians to identify the patients are at risk for committing suicide (63). In our study, the presence of the anhedonia main criterium indicates severe depression, especially when accompanied by feelings of worthless or excessive guilt, and thoughts of death. It is known that the anhedonia, suicidality, and the feelings of worthlessness/excessive guilt criteria may be mainly related to a decrease in central norepinephrine levels (65). Although serotonin has been the most studied neurotransmitter in depression, norepinephrine is also of importance in depressive disorders. An association of specific features and symptoms of depression and a deficiency or dysfunction of certain neurotransmitters has been proposed (65–67): a serotonin deficiency is related to problems such as anxiety, obsessions, and compulsions whereas dysfunctional dopaminergic activity is implicated in problems of motivation and pleasure (66–68). Accordingly, norepinephrine deficiency is associated with increased suicide risk (50, 65, 67).

A limitation of this study is the use of a clinical sample. We choose a clinical sample because it maximizes the prevalence of depression. Although the DSM criteria do not require distinguishing between clinical and non-clinical populations, it is possible to speculate that specific clinical problems may bias both the Hamilton Depression Rating Scale (e.g., irritable bowel syndrome) (69, 70) and the DSM-5 criteria (e.g., fibromyalgia) (71, 72). In addition, the study was done from a sample in Brazil, and some cultural factors could influence the symptoms. Therefore, it would be useful to add other scales to measure depression such as something that the patients could fill out to show if there is a relationship among the multiple instruments (73–75). Another limitation is the lack of power to study sex differences. Although the DSM criteria do not distinguish men from women, men's experiences of depression may be different from women, such as higher rates of anger attacks and aggression in men compared to women (76). It would be of interest to perform a confirmatory analysis using the equations derived from the present data controlling gender and using non- clinical samples.

From a clinical point of view, the present study suggests that somatic rather than cognitive-affective DSM-5 criteria are linked to moderate depression. As moderate depression is associated with adverse cardiovascular outcomes (18, 19), the presence of these somatic symptoms would guide the choice of antidepressants which do not increase cardiac risk. It should be stressed that this recommendation is also important for depressed patients without cardiovascular disease because some antidepressants increase the cardiac risk in individuals without previous cardiac disease (18, 40, 49, 50). As cognitive-affective symptoms are associated with severe depression, the presence of these symptoms increases the need to use antidepressants to prevent suicide (63, 77).

In conclusion, the presence of anhedonia criterium indicates severe depression, especially when accompanied by non-somatic secondary criteria whiles the somatic factors are related to moderate depression. The present study may help the clinical practitioner to infer about depression severity based only on the DSM-5 criteria. The clusters of DSM-5 symptoms exhibited by the patient may help the choice of adequate pharmacological treatments. The correct choice of antidepressants would avoid the use of antidepressants that unnecessarily increase cardiac risk in moderate depression. In addition, when the symptom cluster suggests severe depression, the treatment must focus on the prevention of suicide.

## Author contributions

SS and JT contributed to the overall conception and design of the study. Both authors materially participated in the research and article preparation. JT did the data extraction. SS did the statistical analyses. All authors contributed to interpretation of results and drafting of this manuscript. All authors read and approved the final manuscript.

### Conflict of interest statement

The authors declare that the research was conducted in the absence of any commercial or financial relationships that could be construed as a potential conflict of interest.

## Abbreviations

DM: depressed mood
LI: loss of interest or pleasure
C: diminished ability to think or concentrate
FE: fatigue or loss of energy
FW: feelings of worthlessness or excessive guilt
SU: suicidality
AW: appetite or weight disturbance
PAR: psychomotor agitation or retardation
ND: non-depressed
MD: moderately depressed
SD: severely depressed
DF: discriminant function.
